# Prevalence of mental health symptoms and potential risk factors among Austrian veterinary medicine students

**DOI:** 10.1038/s41598-023-40885-0

**Published:** 2023-08-23

**Authors:** Elke Humer, Viktoria Neubauer, Deianira Brühl, Rachel Dale, Christoph Pieh, Thomas Probst

**Affiliations:** 1 Department for Psychosomatic Medicine and Psychotherapy, University for Continuing Education Krems, 3500 Krems, Austria; 2 Faculty of Psychotherapy Science, Sigmund Freud University Vienna, 1020 Vienna, Austria; 3 Unit for Food Microbiology, Institute of Food Safety, Food Technology, and Veterinary Public Health, Department for Farm Animals and Public Health, University of Veterinary Medicine Vienna, 1210 Vienna, Austria; 4 FFoQSI GmbH-Austrian Competence Centre for Feed and Food Quality, Safety and Innovation, 3430 Tulln, Austria; 5 Division of Psychotherapy, Department of Psychology, University of Salzburg, 5020 Salzburg, Austria

**Keywords:** Health care, Public health, Quality of life, Psychology

## Abstract

Although the poor mental health of veterinarians has been reported in different countries, no data exist on mental health in Austrian veterinary students. This study aimed to provide first data on a broad range of mental health indicators in Austrian veterinary students, compare these data with the Austrian general population, and explore factors associated with poor mental health. A total of 29.1% (n = 430; 85.8% female; mean age: 23.14 ± 3.69 years) of the total Austrian veterinary student population (N = 1477 students; 82.1% females) took part in an online survey conducted from November 2022 to January 2023. Indicators of mental health were symptoms of depression (PHQ-9), anxiety (GAD-7), insomnia (ISI-7), stress (PSS-4), alcohol abuse (CAGE) and disordered eating (SCOFF). Compared to the general Austrian population a higher proportion of veterinary students exceeded the cut-offs for clinically relevant mental health symptoms (*P* < 0.05). A total of 55.3% of participating veterinary students exceeded the cut-off for moderate depressive symptoms, 52.6% for moderate anxiety symptoms, 20.9% for clinically relevant insomnia symptoms, 79.3% for high-stress symptoms, 22.8% for symptoms of alcohol abuse and 38.6% for symptoms of disordered eating. Multivariable logistic regression including several sociodemographic, health behavior, and study-related variables as predictors revealed that mental health symptoms in veterinary students were associated with female gender, older age, low physical activity, high smartphone usage, and desired specification in small animal or wildlife medicine. In conclusion, Austrian veterinary students experience a high mental health burden. The teaching of coping skills and strategies to improve mental hygiene should be implemented in the curricula of veterinary education to improve mental health in the veterinary profession.

## Introduction

Students enrolled in veterinary medicine education programs experience immense pressure, stress, and anxiety from their programs^1,2^. Studies conducted in Germany, the US, and the UK indicate that veterinary students experience enormous strain on mental health with notably higher levels of psychological problems compared to the general population^3–5^ as well as students in other professional programs^6^. Coping poorly with the high demands of the academic training program does not only impair veterinary students` mental health but also their academic performance and this poor coping ability likely persists once veterinarians start practicing^2,7^. The latter is particularly concerning, considering the higher risk of depression and suicide in veterinary practitioners compared to other occupational groups reported in several countries (i.e., the UK, the US, Australia and Austria)^8–11^.

Specific risk factors underlying the increased risk for mental health disorders in this population have barely been investigated so far. Possible reasons for the poor mental health in veterinary students discussed previously include mainly stressors related to the high academic requirements and time pressure associated with veterinary medical education^5,12,13^. The high demands of the study program often brings students to start sacrificing private areas of their lives, such as time for social contacts or sports, to gain more time to study^1^. A decreased study-life balance has been linked to increased levels of depression, anxiety, stress, and substance abuse problems^1,14,15^.

Engaging in regular physical activity is related to improved psychological well-being and a reduced risk of mental health issues^16–18^. Previous studies conducted on Austrian adolescents and adults revealed an association of mental health symptoms with low physical activity^19,20^, whereas specific data on the population of veterinary students are lacking. Besides physical activity, smartphone usage as another important health behavior seems to be associated with mental health. Excessive or problematic smartphone use has been linked to increased levels of stress, anxiety, depression, and other mental health concerns in Austrian adolescents and adults^19,20^.

Besides academic stress and associated changes in health behaviors, also transitional stress seems to impact veterinary students` mental health^21^. Transitional stress in this regard refers to the emotional or psychological strains experienced when students move from high school to university, including stress due to transitioning to a new location, homesickness, a lack of belongingness within their academic community, transportation difficulties, or financial concerns^21,22^. A study investigating self-rated stressors in first-semester veterinary students in the US observed that also financial concerns ranked among the most distressing factors^4^. These findings cannot be directly extrapolated to Austria due to differences in this context. Various factors specific to Austria may influence results, not only related to curricula, but also the financial situation. As an example of differences between countries, while tuition fees for veterinary programs can be substantial in the US, Austria follows a traditional system of tuition-free higher education. Although controversial, previous studies on mental health in practitioners suggest an association between practice type/animal species treated and mental health^8,23–25^. Hence, it appears intriguing to investigate whether there are already differences in mental health among students with varying preferences for different specializations during their studies.

Impaired mental health not only affects subjective well-being and health but also the academic success and dropout rates of veterinary students^1^. Therefore, it is important to elucidate the mental health status of students as well as factors associated with poor mental health status to provide timely support services to vulnerable groups. To date, empirical studies on mental health among veterinary students in Austria are lacking. Thus, the first aim of the current study was to provide novel and valuable baseline data about symptoms of depression, anxiety, insomnia, stress, alcohol abuse, and disordered eating in Austrian veterinary medicine students and to relate these mental health indicators to those measured in the Austrian general population^26^. Based on previous studies we hypothesized a higher prevalence of mental health symptoms in Austrian veterinary medicine students compared to the Austrian general population.

To gain a comprehensive view of potential factors associated with mental health in veterinary medicine students, we assessed specific variables related to the study of veterinary medicine (i.e., study phase, desired specification, working in addition to studying), more general sociodemographic factors (i.e., age, gender, partnership status, country of origin), and health behaviors (i.e., physical activity, smartphone usage) that have been previously found to be associated with mental health in Austrian school students as well as the Austrian general population^19,20,26–28^. As several risk factors are not independent of each other, the aim of our study was not only to examine the association between a single potential risk factor and mental health but also to investigate the independent contribution of each independent variable in predicting the prevalence of mental health disorders by adjusting for the other variables. Based on the available literature we hypothesized an association of the odds for exceeding cut-offs for clinically relevant mental health symptoms with the following variables: female gender, not born in Austria, older age, being single, employment alongside the study, not meeting recommendations for physical activity, spending excessive time on the smartphone. We had no hypothesis regarding the desired specification.

The following research questions were addressed:

1. How do symptoms of depression, anxiety, insomnia, stress, alcohol abuse, and disordered eating in Austrian veterinary medicine students compare to those measured in the Austrian general population?
2. What is the prevalence of symptoms of depression, anxiety, insomnia, stress, alcohol abuse, and disordered eating among Austrian veterinary medicine students?
3. What are the potential risk factors associated with symptoms of depression, anxiety, insomnia, stress, alcohol abuse, and disordered eating among veterinary medicine students?

## Results

### Study sample characteristics

In total, N = 430 (including gender-diverse participants) veterinary medicine students participated (response rate: 29.11%). Sociodemographic, health behavior and study characteristics are summarized in Table 1. They were 23.14 ± 3.69 years old and 85.8% were female (compared to 82.1% in the population of students at the University of Veterinary Medicine Vienna). The majority (67.2%) were born in Austria and they had on average studied 5.74 ± 3.69 semesters. In addition to being a student, 39.8% reported working for an average of 11.30 ± 6.62 h per week. The highest proportion (44%) stated to be mainly interested in small animal medicine, followed by equine (18.1%) and ruminant (16.5%) medicine. Nearly half of the students (48.6%) were physically active less than 3 days per week and about two-thirds (66.4%) spent at least 3 h per day on their smartphones.

**Table 1:** Study sample characteristics (N = 430).

Variable	
Gender	
Female, % (N)	85.8 (369)
Male, % (N)	13.5 (58)
Diverse, % (N)	0.7 (3)
Age in years, M (SD)	23.14 (3.69)
Partnership status	
Single, % (N)	49.3 (212)
Partnership, % (N)	50.7 (218)
Country of origin	
Austria, % (N)	67.2 (289)
Other countries, % (N)	32.8 (141)
Semester, M (SD)	5.74 (3.69)
Employment	
Yes, % (N)	39.8 (171)
No, % (N)	60.2 (259)
Intended specialization	
Small animal medicine, % (N)	44.0 (189)
Ruminant medicine, % (N)	16.5 (71)
Equine medicine, % (N)	18.1 (78)
Zoo and wildlife medicine), % (N)	14.2 (61)
Poultry and swine medicine, % (N)	1.9 (8)
Reproductive technology, % (N)	0.5 (2)
Laboratory animal medicine, % (N)	2.8 (12)
Food science, public veterinary, and healthcare, % (N)	2.1 (9)
Physical activity for at least 60min/per day	
0 day/week, % (N)	17.4 (75)
1 day/week, % (N)	14.7 (63)
2 day/week, % (N)	16.5 (71)
3 day/week, % (N)	17.2 (74)
4 day/week, % (N)	12.6 (54)
5 day/week, % (N)	9.3 (40)
6 day/week, % (N)	4.4 (19)
7 day/week, % (N)	7.9 (34)
Smartphone usage	
< 1 h/day, % (N)	4.0 (17)
1–2 h/day, % (N)	29.8 (128)
3–4 h/day, % (N)	50.0 (215)
5–6 h/day, % (N)	12.6 (54)
7–8 h/day, % (N)	2.6 (11)
> 8 h/day, % (N)	1.2 (5)

### Mental health indicators in veterinary medicine students 

Descriptive analyses revealed that 55.3% of participants (including gender-diverse participants) exceeded the cut-off for moderate depressive symptoms, 52.6% for moderate anxiety symptoms, 20.9% for clinically relevant insomnia symptoms, 79.3% for high-stress, 22.8% for symptoms of alcohol abuse, and 38.6% for symptoms of disordered eating.

### Comparison of mental health indicators in veterinary medicine students with the general population

Compared with the general population a higher proportion of veterinary students (excluding gender-diverse participants) scored above the cut-offs for clinically relevant mental health problems (depressive symptoms: 55.0% compared to 28.0%, anxiety symptoms: 52.7% compared to 15.9%, insomnia symptoms: 21.1% compared to 14.3%, high stress: 79.2% compared to 55.9%, symptoms of alcohol abuse: 22.7% compared to 17.9%, symptoms of disordered eating: 38.6% compared to 25.7%; *P* ≤ 0.035; Table 2).

**Table 2:** Proportion of veterinary students and a representative sample of the Austrian general population exceeding the cut-off scores for symptoms of depression/anxiety/insomnia/stress/alcohol abuse and disordered eating by group (n = 1438).

Variable		Group		Statistics
		General population (n = 1011)	Veterinary students (n = 427)	
Depression	%	28.0	55.0	χ2 (1) = 95.27; *P* < 0.001
	n	283	235	
Anxiety	%	15.9	52.7	χ2 (1) = 206.67; *P* < 0.001
	n	161	225	
Insomnia	%	14.3	21.1	χ2 (1) = 9.96; *P* = 0.002
	n	145	90	
High stress	%	55.9	79.2	χ2 (1) = 69.17; *P* < 0.001
	n	565	338	
Alcohol abuse	%	17.9	22.7	χ2 (1) = 4.46; *P* = 0.035
	n	181	97	
Disordered eating	%	25.7	38.6	χ2 (1) = 24.09; *P* < 0.001
	n	260	165	

*P P*-values (2-tailed), *χ2* Chi-squared-test, *Depression* ≥ 10 points on the Patient Health Questionnaire 9 scale, *Anxiety* ≥ 10 points on the Generalised Anxiety Disorder 7 scale, *Insomnia* ≥ 15 on the 7-item Insomnia Severity Index, *High stress* ≥ 6 points on the Perceived Stress Scale 4, *Alcohol abuse* ≥ 2 on the 4-item CAGE questionnaire, *Disordered eating* ≥ 2 on the 5-item SCOFF questionnaire.

**Figure 1:**
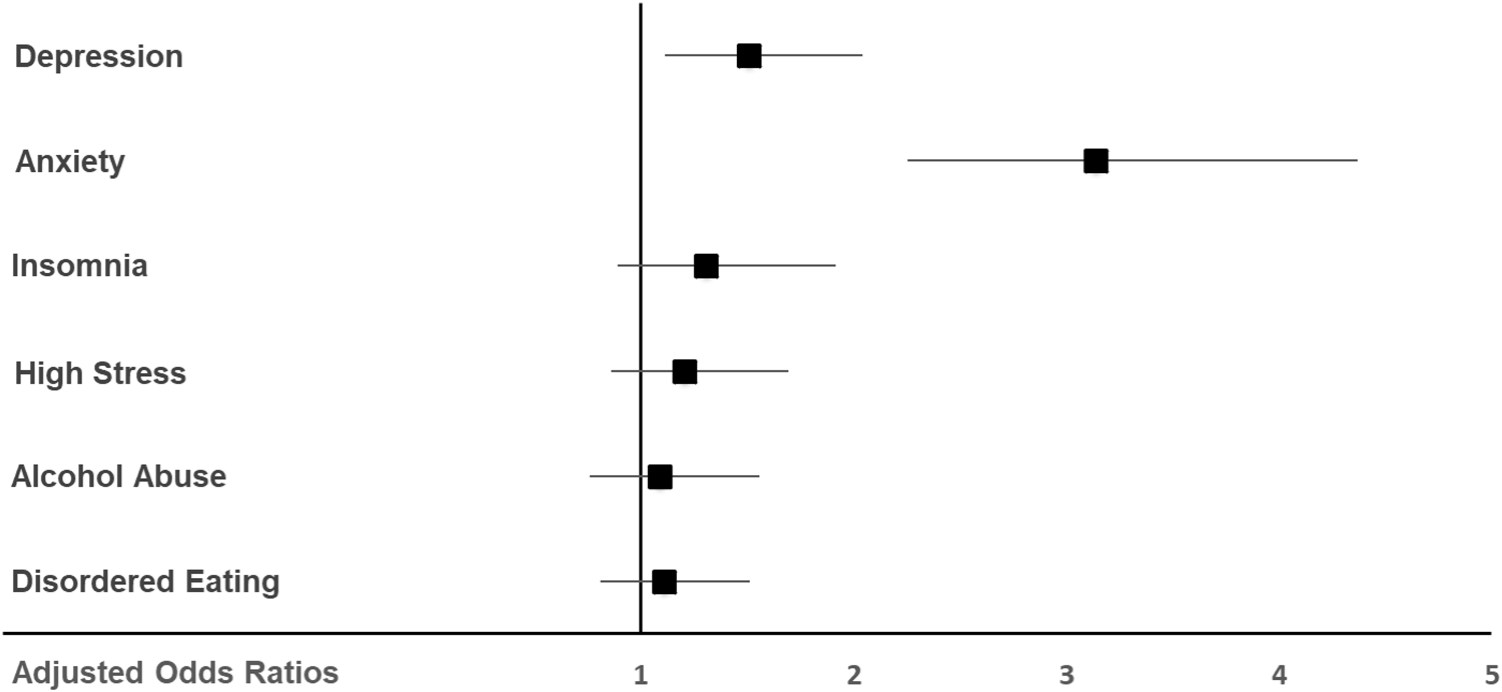
Adjusted odds ratios for clinically relevant symptoms of depression, anxiety, insomnia, stress, alcohol abuse, and disordered eating in Austrian veterinary medicine students (n = 427) compared to a representative sample of the general population (n = 1011), after controlling for gender and age (1 = no difference, > 1 = veterinary students have more mental health symptoms than the general population).

As mental health symptoms are more prevalent in women compared to men and in younger compared to older adults in the representative sample of the general population^26^, potential differences between both groups were also assessed taking gender and age as predictors into account. Symptoms of depression (aOR 1.51, 95% CI 1.11, 2.04; *P* = 0.008) and anxiety (aOR 3.14, 95% CI 2.25, 4.37; *P* < 0.001) were more likely to be observed in veterinary students compared to the general population (Fig. 1). The odds for experiencing clinically relevant insomnia symptoms (aOR 1.30, 95% CI 0.89, 1.91; *P* = 0.174), high-stress levels (aOR 1.21, 95% CI 0.86, 1.69; *P* = 0.274), symptoms of alcohol abuse (aOR 1.09, 95% CI 0.76, 1.56; *P* = 0.647) and symptoms of disordered eating (aOR 1.11, 95% CI 0.81, 1.51; *P* = 0.521) did not differ significantly between both groups.

Male gender decreased the odds for symptoms of depression (aOR 0.65, 95% CI 0.50, 0.84; *P* = 0.001), anxiety (aOR 0.62, 95% CI 0.46, 0.83; *P* = 0.001), high stress (aOR 0.70, 95% CI 0.55, 0.89; *P* = 0.004), and disordered eating (aOR 0.73, 95% CI 0.56, 0.94; *P* = 0.016), but increased the odds for signs of alcohol abuse (aOR 1.87, 95% CI 1.40, 2.51; *P* < 0.001). The odds for experiencing clinically relevant symptoms of insomnia (aOR 0.91, 95% CI 0.66, 1.24; *P* = 0.550) were not associated with gender.

Increasing age went along with reduced odds for exceeding the cut-offs of clinically relevant symptoms of depression (aOR 0.97, 95% CI 0.96, 0.98; *P* < 0.001), anxiety (aOR 0.98, 95% CI 0.97, 0.99; *P* < 0.001), high stress (aOR 0.97, 95% CI 0.96, 0.98; *P* < 0.001), alcohol abuse (aOR 0.98, 95% CI 0.97, 0.99; *P* < 0.001) and disordered eating (aOR 0.98, 95% CI 0.97, 0.99; *P* < 0.001), but was not associated with the odds for experiencing clinically relevant symptoms of insomnia (aOR 0.99, 95% CI 0.98, 1.00; *P* = 0.255).

### Univariate associations of sociodemographic factors, health behaviors, and study-related variables with mental health indicators in veterinary medicine students

#### Sociodemographic variables

Chi-squared-tests showed a higher proportion of female students scoring above the cut-off for clinically relevant symptoms of anxiety (55.8% compared to 32.8%) and disordered eating (41.2% compared to 22.4%) compared to male students (*P* ≤ 0.006; Table 3). On the contrary, alcohol abuse symptoms were more prevalent among male (41.4%) than female (19.8%) students (*P* < 0.001).

**Table 3:** Proportion of participants exceeding the cut-off scores for symptoms of depression/anxiety/insomnia/stress/alcohol abuse/disordered eating by gender (n = 427).

Variable		Gender		Statistics
		Female (n = 369)	Male (n = 58)	
Depression	%	56.1	48.3	χ2 (1) = 1.24; *P* = 0.266
	n	207	28	
Anxiety	%	**55.8**	**32.8**	**χ2 (1) = 10.70; *P* = 0.001**
	n	**206**	**19**	
Insomnia	%	21.1	20.7	χ2 (1) = 0.01; *P* = 0.938
	n	78	12	
High stress	%	79.7	75.9	χ2 (1) = 0.44; *P* = 0.506
	n	294	44	
Alcohol abuse	%	**19.8**	**41.4**	**χ2 (1) = 13.32; *P* < 0.001**
	n	**73**	**24**	
Disordered eating	%	**41.2**	**22.4**	**χ2 (1) = 7.56; *P* = 0.006**
	n	**152**	**13**	

*P P*-values (2-tailed), *χ2* Chi-squared-test, *Depression* ≥ 10 points on the Patient Health Questionnaire 9 scale, *Anxiety* ≥ 10 points on the Generalised Anxiety Disorder 7 scale, *Insomnia* ≥ 15 on the 7-item Insomnia Severity Index, *High stress* ≥ 6 points on the Perceived Stress Scale 4, *Alcohol abuse* ≥ 2 on the 4-item CAGE questionnaire, *Disordered eating* ≥ 2 on the 5-item SCOFF questionnaire.

Significant values are given in bold.

Age was significantly associated with symptoms of depression, insomnia, and alcohol abuse (Table 4). Students older than 26 years had the highest prevalence of depressive symptoms (76.3%), differing significantly from those aged between 21 and 23 years (48.2%; *P* = 0.001). Insomnia symptoms were more frequent in students older than 26 years compared to all other age groups (*P* = 0.01). Symptoms of alcohol abuse were more prevalent in 24–26-year-old students compared to 21- to 23-year-old students (*P* = 0.021).

**Table 4:** Proportion of participants exceeding the cut-off scores for symptoms of depression/anxiety/insomnia/stress/alcohol abuse/disordered eating by age (n = 427).

Variable		Age (in years)				Statistics
		18–20 (n = 96)	21–23 (n = 168)	24–26 (n = 104)	≥ 27 (n = 59)	
Depression	%	**50.0**	**48.2**	**58.7**	**76.3**	**χ2 (3) = 15.44; *P* = 0.001**
	n	**48**	**81**	**61**	**45**	
Anxiety	%	51.0	47.0	55.8	66.1	χ2 (3) = 6.92; *P* = 0.074
	n	49	79	58	39	
Insomnia	%	**19.8**	**16.7**	**20.2**	**37.3**	**χ2 (3) = 11.43; *P* = 0.010**
	n	**19**	**28**	**21**	**22**	
High stress	%	83.3	72.6	83.7	83.1	χ2 (3) = 7.18; P = 0.066
	n	80	122	87	49	
Alcohol abuse	%	**18.8**	**17.3**	**31.7**	**28.8**	**χ2 (3) = 9.77; *P* = 0.021**
	n	**18**	**29**	**33**	**17**	
Disordered eating	%	38.5	41.1	30.8	45.8	χ2 (3) = 4.40; *P* = 0.221
	n	37	69	32	27	

*P P*-values (2-tailed), *χ2* Chi-squared-test, *Depression* ≥ 10 points on the Patient Health Questionnaire 9 scale, *Anxiety* ≥ 10 points on the Generalised Anxiety Disorder 7 scale, *Insomnia* ≥ 15 on the 7-item Insomnia Severity Index, *High stress* ≥ 6 points on the Perceived Stress Scale 4, *Alcohol abuse* ≥ 2 on the 4-item CAGE questionnaire, *Disordered eating* ≥ 2 on the 5-item SCOFF questionnaire.

Significant values are given in bold.

The country of origin was not associated with the prevalence of mental health symptoms (Table 5).

**Table 5:** Proportion of participants exceeding the cut-off scores for symptoms of depression/anxiety/insomnia/stress/alcohol abuse/disordered eating by country of origin (n = 427).

Variable		Country		Statistics
		Austria (n = 286)	Others (n = 141)	
Depression	%	53.1	58.9	χ2 (1) = 1.25; *P* = 0.264
	n	152	83	
Anxiety	%	51.4	55.3	χ2 (1) = 0.58; *P* = 0.445
	n	147	78	
Insomnia	%	19.9	23.4	χ2 (1) = 0.69; *P* = 0.408
	n	57	33	
High stress	%	80.1	77.3	χ2 (1) = 0.44; *P* = 0.508
	n	229	109	
Alcohol abuse	%	22.4	23.4	χ2 (1) = 0.06; *P* = 0.812
	n	64	33	
Disordered eating	%	38.5	39.0	χ2 (1) = 0.01; *P* = 0.913
	n	110	55	

*P*: *P* -values (2-tailed), χ2: Chi-squared-test; Depression: ≥ 10 points on the Patient Health Questionnaire 9 scale; Anxiety: ≥ 10 points on the Generalised Anxiety Disorder 7 scale; Insomnia: ≥ 15 on the 7-item Insomnia Severity Index; High Stress: ≥ 6 points on the Perceived Stress Scale 4; Alcohol Abuse: ≥ 2 on the 4-item CAGE questionnaire; Disordered Eating: ≥ 2 on the 5-item SCOFF questionnaire.

Students living in a partnership had higher rates of anxiety symptoms (58.7%) compared to those who were single (46.4%; *P* = 0.011; Table 6).

**Table 6:** Proportion of participants exceeding the cut-off scores for symptoms of depression/anxiety/insomnia/stress/alcohol abuse/disordered eating by partnership status (n = 427).

Variable		Partnership status		Statistics
		Single (n = 209)	Partnership (n = 218)	
Depression	%	50.2	59.6	χ2 (1) = 3.81; *P* = 0.051
	n	105	130	
Anxiety	%	**46.4**	**58.7**	**χ2 (1) = 6.48; *P* = 0.011**
	n	**97**	**128**	
Insomnia	%	18.2	23.9	χ2 (1) = 2.06; *P* = 0.151
	n	38	52	
High stress	%	79.4	78.9	χ2 (1) = 0.02; *P* = 0.893
	n	166	172	
Alcohol abuse	%	20.1	25.2	χ2 (1) = 1.60; *P* = 0.206
	n	42	55	
Disordered eating	%	39.2	38.1	χ2 (1) = 0.06; *P* = 0.805
	n	82	83	

*P*: *P* -values (2-tailed; χ2: Chi-squared-test; Depression: ≥ 10 points on the Patient Health Questionnaire 9 scale; Anxiety: ≥ 10 points on the Generalised Anxiety Disorder 7 scale; Insomnia: ≥ 15 on the 7-item Insomnia Severity Index; High Stress: ≥ 6 points on the Perceived Stress Scale 4; Alcohol Abuse: ≥ 2 on the 4-item CAGE questionnaire; Disordered Eating: ≥ 2 on the 5-item SCOFF questionnaire.

Significant values are given in bold.

#### Health behaviors

Physical activity of at least 3 days per week for at least one hour was associated with lower rates of symptoms of anxiety (45.7% compared to 60.2%) and alcohol abuse (16.3% compared to 29.6%) than spending less than 3 days per week physically active (*P* ≤ 0.004; Table 7). Students who spent ≥ 3h/d on their smartphones had higher rates of symptoms of disordered eating (43.3%) compared to those spending up to 2 h/d on their smartphones (29.7%; *P* = 0.006; Table 8).

**Table 7:** Proportion of participants exceeding the cut-off scores for symptoms of depression/anxiety/insomnia/stress/alcohol abuse/disordered eating by physical activity (n = 427).

Variable		Physical activity		Statistics
		≤ 2 day/week (n = 206)	≥ 3 day/week (n = 221)	
Depression	%	59.7	50.7	χ2 (1) = 3.51; *P* = 0.061
	n	123	112	
Anxiety	%	**60.2**	**45.7**	**χ2 (1) = 8.98; *P* = 0.004**
	n	**124**	**101**	
Insomnia	%	20.9	21.3	χ2 (1) = 0.01; *P* = 0.921
	n	43	47	
High stress	%	82.0	76.5	χ2 (1) = 2.00; *P* = 0.157
	n	169	169	
Alcohol abuse	%	**29.6**	**16.3**	**χ2 (1) = 10.78; *P* = 0.001**
	n	**61**	**36**	
Disordered eating	%	38.8	38.5	χ2 (1) = 0.01; *P* = 0.937
	n	80	85	

*P*: *P* -values (2-tailed), χ2: Chi-squared-test; Depression: ≥ 10 points on the Patient Health Questionnaire 9 scale; Anxiety: ≥ 10 points on the Generalised Anxiety Disorder 7 scale; Insomnia: ≥ 15 on the 7-item Insomnia Severity Index; High Stress: ≥ 6 points on the Perceived Stress Scale 4; Alcohol Abuse: ≥ 2 on the 4-item CAGE questionnaire; Disordered Eating: ≥ 2 on the 5-item SCOFF questionnaire.

Significant values are given in bold.

**Table 8:** Proportion of participants exceeding the cut-off scores for symptoms of depression/anxiety/insomnia/stress/alcohol abuse/disordered eating by smartphone usage (n = 427).

Variable		Smartphone usage		Statistics
		≤ 2 h/day (n = 145)	≥ 3 h/day (n = 282)	
Depression	%	51.7	56.7	χ2 (1) = 0.97; *P* = 0.324
	n	75	160	
Anxiety	%	47.6	55.3	χ2 (1) = 2.30; *P* = 0.130
	n	69	156	
Insomnia	%	21.4	20.9	χ2 (1) = 0.01; *P* = 0.913
	n	31	59	
High stress	%	76.6	80.5	χ2 (1) = 0.90; *P* = 0.342
	n	111	227	
Alcohol abuse	%	24.1	22.0	χ2 (1) = 0.25; *P* = 0.615
	n	35	62	
Disordered eating	%	**29.7**	**43.3**	**χ2 (1) = 7.48; *P* = 0.006**
	n	**43**	**122**	

*P P*-values (2-tailed), *χ2* Chi-squared-test, *Depression* ≥ 10 points on the Patient Health Questionnaire 9 scale, *Anxiety* ≥ 10 points on the Generalised Anxiety Disorder 7 scale, *Insomnia* ≥ 15 on the 7-item Insomnia Severity Index, *High stress* ≥ 6 points on the Perceived Stress Scale 4, *Alcohol abuse* ≥ 2 on the 4-item CAGE questionnaire, *Disordered eating* ≥ 2 on the 5-item SCOFF questionnaire.

Significant values are given in bold.

#### Study-related variables

Students in the last phase of their study had the highest rates of symptoms of alcohol abuse (34.9%; *P* = 0.011; Table 9).

**Table 9:** Proportion of participants exceeding the cut-off scores for symptoms of depression/anxiety/insomnia/stress/alcohol abuse/disordered eating by study phase (n = 427).

Variable		Study phase			Statistics
		1st (n = 181)	2nd (n = 160)	3rd (n = 86)	
Depression	%	51.9	56.3	59.3	χ2 (2) = 1.43; *P* = 0.489
	n	94	90	51	
Anxiety	%	52.5	51.9	54.7	χ2 (2) = 0.18; *P* = 0.915
	n	95	83	47	
Insomnia	%	18.2	23.1	23.3	χ2 (2) = 1.53; *P* = 0.465
	n	33	37	20	
High stress	%	76.8	80.0	82.6	χ2 (2) = 1.28; *P* = 0.526
	n	139	128	71	
Alcohol abuse	%	**19.3**	**20.0**	**34.9**	**χ2 (2) = 9.10; *P* = 0.011**
	n	**35**	**32**	**30**	
Disordered eating	%	37.0	38.8	41.9	χ2 (2) = 0.58; *P* = 0.749
	n	67	62	36	

*P P*-values (2-tailed), *χ2* Chi-squared-test, *Depression* ≥ 10 points on the Patient Health Questionnaire 9 scale, *Anxiety* ≥ 10 points on the Generalised Anxiety Disorder 7 scale, *Insomnia* ≥ 15 on the 7-item Insomnia Severity Index, *High stress* ≥ 6 points on the Perceived Stress Scale 4, *Alcohol abuse* ≥ 2 on the 4-item CAGE questionnaire, *Disordered eating* ≥ 2 on the 5-item SCOFF questionnaire.

Significant values are given in bold.

Being employed next to the study activities was not associated with the investigated mental health indicators (Table 10).

**Table 10:** Proportion of participants exceeding the cut-off scores for symptoms of depression/anxiety/insomnia/stress/alcohol abuse/disordered eating by employment status (n = 427).

Variable		Employment		Statistics
		Yes (n = 170)	No (n = 257)	
Depression	%	55.9	54.5	χ2 (1) = 0.08; *P* = 0.775
	n	95	140	
Anxiety	%	48.8	55.3	χ2 (1) = 1.70; *P* = 0.193
	n	83	142	
Insomnia	%	24.7	18.7	χ2 (1) = 2.24; *P* = 0.135
	n	42	48	
High stress	%	77.6	80.2	χ2 (1) = 0.39; *P* = 0.532
	n	132	206	
Alcohol abuse	%	24.1	21.8	χ2 (1) = 0.32; *P* = 0.574
	n	41	56	
Disordered eating	%	41.2	37.0	χ2 (1) = 0.77; *P* = 0.383
	n	70	95	

*P P*-values (2-tailed), *χ2* Chi-squared-test, *Depression* ≥ 10 points on the Patient Health Questionnaire 9 scale, *Anxiety* ≥ 10 points on the Generalised Anxiety Disorder 7 scale, *Insomnia* ≥ 15 on the 7-item Insomnia Severity Index, *High stress* ≥ 6 points on the Perceived Stress Scale 4, *Alcohol abuse* ≥ 2 on the 4-item CAGE questionnaire, *Disordered eating* ≥ 2 on the 5-item SCOFF questionnaire.

The desired specification was associated with different prevalences of depressive symptoms and anxiety symptoms (*P* ≤ 0.002; Table 11). The highest rates of depressive symptoms were observed in students who intended to focus on zoo and wildlife medicine or small animal medicine, differing from those interested in equine and ruminant medicine (*P* < 0.001). The highest rates of anxiety symptoms were observed in students who intended to focus on zoo and wildlife medicine or small animal medicine, differing from those interested in ruminant medicine (*P* = 0.002).

**Table 11:** Proportion of participants exceeding the cut-off scores for symptoms of depression/anxiety/insomnia/stress/alcohol abuse/disordered eating by (desired) specification (n = 427).

Variable		Specification					Statistics
		Small animal medicine (n = 188)	Ruminant medicine (n = 70)	Equine medicine (n = 77)	Zoo and wildlife medicine (n = 61)	Others(n = 31)	
Depression	%	**61.2**	**40.0**	**42.9**	**70.5**	**51.6**	**χ2 (4) = 19.90; *P* = 0.001**
	n	**115**	**28**	**33**	**43**	**16**	
Anxiety	%	**59.0**	**37.1**	**45.5**	**65.6**	**41.9**	**χ2 (4) = 16.50; *P* = 0.002**
	n	**111**	**26**	**35**	**40**	**13**	
Insomnia	%	25.5	15.7	15.6	19.7	22.6	χ2 (4) = 4.96; *P* = 0.291
	n	48	11	12	12	7	
High stress	%	80.3	74.3	80.5	86.9	64.5	χ2 (4) = 7.48; *P* = 0.112
	n	151	52	62	53	20	
Alcohol abuse	%	19.7	28.6	20.8	24.6	29.0	χ2 (4) = 3.35; *P* = 0.502
	n	37	20	16	15	9	
Disordered eating	%	38.8	27.1	42.9	42.6	45.2	χ2 (4) = 5.45; *P* = 0.244
	n	73	19	33	26	14	

*P P* -values (2-tailed), *χ2* Chi-squared-test, *Depression* ≥ 10 points on the patient health questionnaire 9 scale, *Anxiety* ≥ 10 points on the Generalised Anxiety Disorder 7 scale, *Insomnia* ≥ 15 on the 7-item Insomnia Severity Index, *High stress* ≥ 6 points on the Perceived Stress Scale 4, *Alcohol abuse* ≥ 2 on the 4-item CAGE questionnaire, *Disordered eating* ≥ 2 on the 5-item SCOFF questionnaire.

Significant values are given in bold.

### Multivariable analyses of the association of sociodemographic factors, health behaviors, and study-related variables with mental health indicators in veterinary medicine students

Detailed results of the logistic regression analyses are summarized in Suppl. Tables S1–S6.

#### Sociodemographic variables

**Figure 2:**
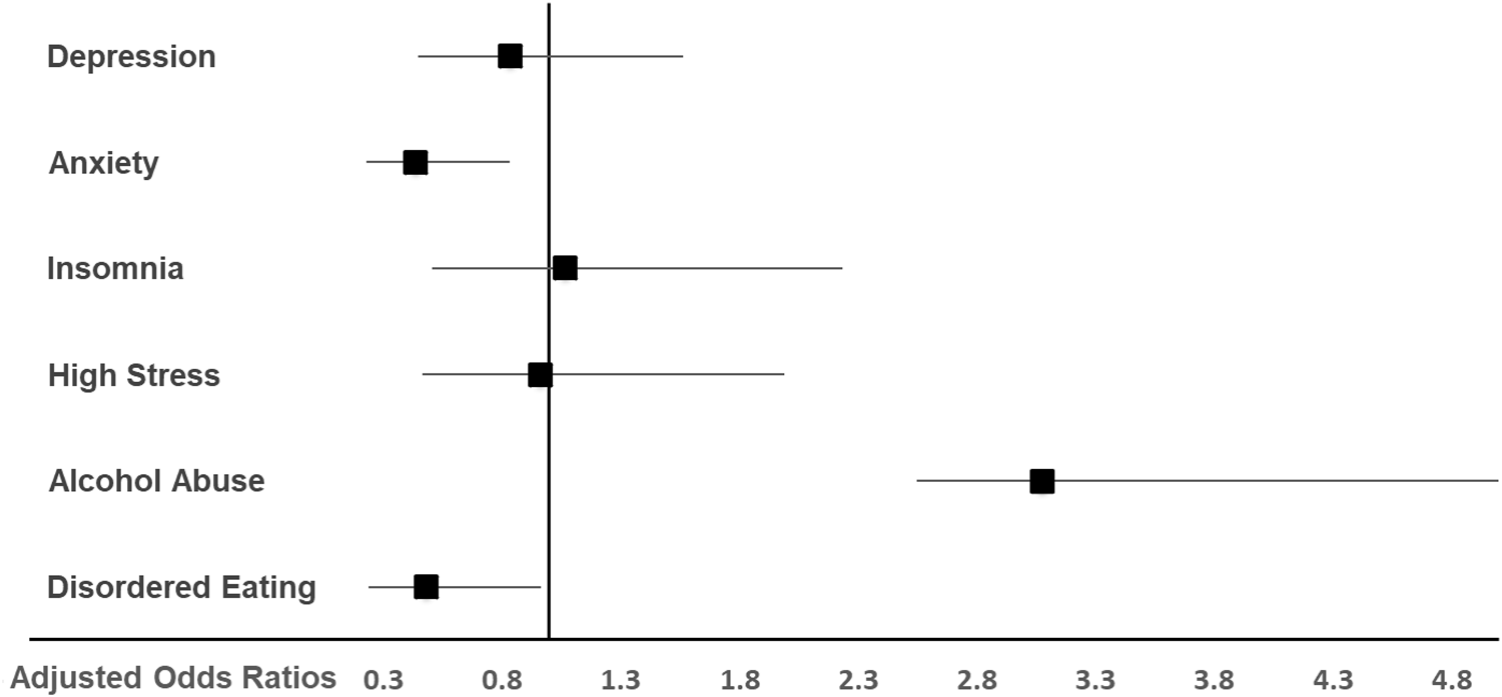
Adjusted odds ratios for clinically relevant symptoms of depression, anxiety, insomnia, stress, alcohol abuse and disordered eating in male (n = 58) compared to female (n = 369) veterinary medicine students (1 = no difference, > 1 = male students have more mental health symptoms than female students).

As depicted in Fig. 2, the male gender decreased the odds for anxiety symptoms (aOR: 0.44; 95% CI 0.23, 0.83;* P* = 0.012) and symptoms of disordered eating (aOR: 0.48; 95% CI 0.24, 0.97;* P* = 0.040), whereas it increased the odds for symptoms of alcohol abuse (aOR: 3.08; 95% CI 1.59, 5.94;* P* = 0.001).

With increasing age, the odds for depressive symptoms (aOR: 1.13; 95% CI 1.05, 1.23;* P* = 0.002) and anxiety symptoms (aOR: 1.08; 95% CI 1.01, 1.16;* P* = 0.026) increased.

No association of partnership status and country of origin with the investigated mental health indicators in veterinary students was observed.

#### Health behaviors

**Figure 3:**
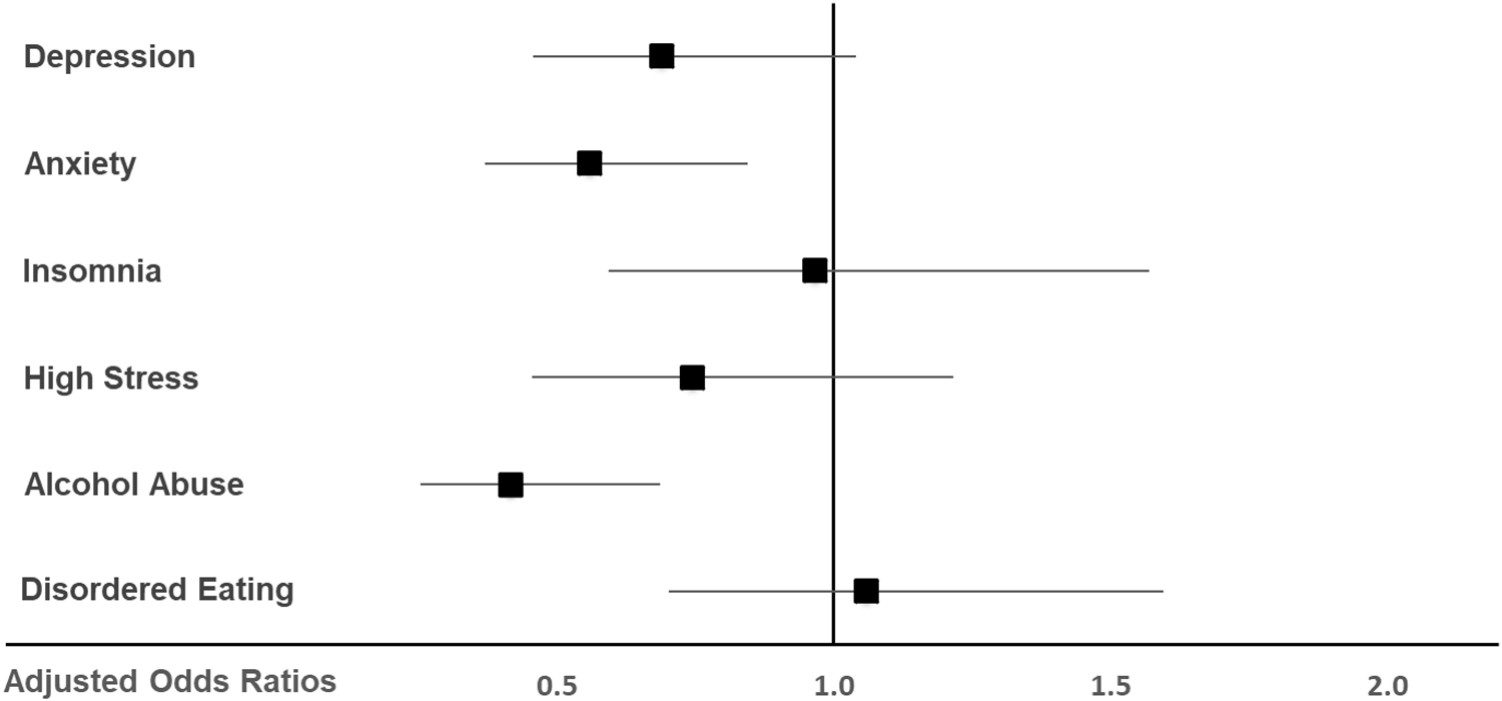
Adjusted odds ratios for clinically relevant symptoms of depression, anxiety, insomnia, stress, alcohol abuse and disordered eating in veterinary medicine students being physically active at least 3 days per week (n = 221) compared to those being physically active up to 2 days per week (n = 206), (1 = no difference, < 1 = physically active students have less mental health symptoms than physically inactive students).

Physical activity (defined as being physically active for at least 60 min per day on at least three days per week) was associated with reduced risk for exceeding the cut-offs for clinically relevant anxiety symptoms (aOR: 0.56; 95% CI 0.37; 0.84;* P* = 0.006) and alcohol abuse symptoms (aOR: 0.42; 95% CI 0.25; 0.69;* P* = 0.001) as depicted in Fig. 3.

Using the smartphone for at least 3 h per day increased the odds of experiencing symptoms of disordered eating (aOR: 1.85; 95% CI 1.18; 2.91;* P* = 0.007).

#### Study-related variables

Among the variables related to the study (study phase, specification, employment status), only the desired specification was found to be associated with the odds of experiencing mental health symptoms. More specifically, desired specification in ruminant medicine compared to small animal medicine reduced the odds for symptoms of depression (aOR: 0.42; 95% CI 0.23, 0.76;* P* = 0.004) and anxiety (aOR: 0.46; 95% CI 0.25, 0.84;* P* = 0.011). Similarly, desired specification in equine medicine compared to small animal medicine reduced the odds for symptoms of depression (aOR: 0.43; 95% CI 0.24, 0.75;* P* = 0.003) and anxiety (aOR: 0.52; 95% CI 0.29, 0.91;* P* = 0.021). Students who stated that they wish to specify in other fields (i.e., poultry and swine medicine, reproductive technology, laboratory animal medicine, food science, public veterinary, and health care) compared to those who wished to specify in small animal medicine had lower odds for experiencing high stress (aOR: 0.42; 95% CI 0.18, 0.99;* P* = 0.048).

## Discussion

The current study explored the mental health of Austrian veterinary medicine students and factors associated with mental health indicators. There are two major findings of this study. First, results indicate that Austrian veterinary medicine students experience a high mental health burden, especially with symptoms of depression and anxiety exceeding those of the general population. Second, older students and female students are significantly more burdened, physical activity and lower time spent with the smartphone has a positive association with mental health, and among study-related variables, the desired specification was found to be associated with mental health outcomes. In the following, we present a more detailed discussion of the major findings, with a focus on addressing the research questions formulated in the introduction.

### Comparison of mental health symptoms in Austrian veterinary medicine students compared to the Austrian general population

In line with results from other countries, as reviewed systematically by Platt et al.^29^, the findings at hand indicate that Austrian veterinary medicine students experience a high mental health burden, especially with symptoms of depression and anxiety exceeding those of the general population. Still, it must be considered that the survey on veterinary students was conducted in winter 2022/23, whereas the data on the general population were collected in spring 2022. At the time of study in the general population, COVID-19 containment efforts were mainly limited to the mask mandates in critical infrastructures and the requirement of proof of low epidemic risk (i.e., proof to be vaccinated, recovered, or tested) upon entering Austria^30^. During the time of the survey of the veterinary students, only minimal COVID-19 restrictions were in place, such as mask mandates in healthcare facilities. Although most COVID-19 measures were lifted already before the survey in the general population, it can be assumed that during the survey of the veterinary students, pandemic-related issues were of less importance to the participants. Also, a few weeks before the survey in the general population several new crises and issues emerged (i.e., the Russian-Ukraine war, high inflation rates, and the gas/energy crisis)^31^. Therefore, the different circumstances at both time points might confound the results. In future studies comparing the mental health of veterinary students with other populations (e.g., general population or non-veterinary students), a better synchronization of the study times should be aimed for.

### Prevalence of mental health symptoms among Austrian veterinary medicine students

The current data, relying on 29.1% of the total population of Austrian veterinary students, indicate that Austrian veterinary medicine students experience a high mental health burden.

A previous study conducted on German veterinary medicine students from November 2018 to April 2019 observed clinically relevant depression (assessed with the same instrument, the PHQ-9) in 45.9% of the veterinary students^5^. Studies conducted in the US using the same scale observed a prevalence of 33.9% for depression in veterinary students^32^. Thus, it seems that the prevalence of depression is even higher (55.3%) in Austrian veterinary students. Similarly, the levels of anxiety assessed with the GAD-7 ranged at 36.2% in US students^32^, while in the study at hand, 52.6% of the students scored above the cut-off for clinically relevant anxiety symptoms. Yet, the studies from other countries were conducted before the COVID-19 pandemic and other crises, which could have influenced the higher proportion of mental health problems in the Austrian sample*.*

While several previous studies investigated symptoms of depression and anxiety in veterinary students, less is known about the occurrence of hazardous drinking and disordered eating. A study on 509 UK students observed that 10% of veterinary students suffered from eating disorders^3^. However, a direct comparison to the 38.6% of students exceeding the cut-off for symptoms of disordered eating in the study at hand is not possible, due to the usage of different assessment tools. In line with previous studies symptoms of disordered eating were more frequent in female compared to male students of other faculties^33,34^ and increased with time spent on the smartphone^19^. For alcohol abuse, the 22.8% measured with the CAGE in the present study is in line with rates of 25% assessed with the Alcohol Use Disorder Identification Test (AUDIT) of the WHO in US veterinary students^32^. Similar occurrence of problematic alcohol consumption in veterinary students compared to the general population as well as more male students reporting hazardous alcohol consumption compared to female students have been reported previously^35^. Analyses of drinking motives in veterinary students revealed that alcohol was primarily used as a coping mechanism to regulate emotions^35^.

### Potential risk factors associated with mental health symptoms among veterinary medicine students

The current data reveal that older students and female students are significantly more burdened, physical activity and lower time spent with the smartphone have a positive association with mental health, and among study-related variables, the desired specification was found to be associated with mental health outcomes.

Results on increased mental health burden in females compared to males (except for problematic alcohol consumption) are in line with previous studies conducted on veterinary students^32,36^. Data on the association of older age with higher odds for mental health symptoms are more difficult to interpret. As data obtained from the general Austrian population demonstrates an association of mental health symptoms with younger age, poor academic performance can be speculated to be a relevant factor behind the observed association of older age and poor mental health in veterinary students. As the duration of studying in years or other relevant data on study success (such as the number of exams failed, the number of lost semesters) was not assessed, it is not possible to conclude whether older students were off course. This aspect needs to be evaluated in more depth in further studies.

Higher mental health burdens in individuals with low physical activity and those spending more time with their smartphones are following findings in the Austrian general population^18,19^ as well as school students aged between 14 and 20^20^. Thus, the present results further underscore the need to promote health behaviors (i.e., physical activity) as an adaptive coping strategy in veterinary students to mitigate mental health problems.

Among study-related variables, only the desired specification was associated with mental health symptoms. Previous results on the association between the type of practice and mental health have been controversial^23^, with some studies pointing at a higher mental health burden in veterinarians practicing exotic^25^ or small animal medicine^24,25^, while others found no association between mental health and practice type^8^. Overall, results are difficult to compare, as large variations occur among countries and universities, not only related to the curricula, but also related to the financial situation. As an example of differences between countries, while tuition fees for veterinary programs can be substantial in the US, Austria follows a traditional system of tuition-free higher education. These differences might be one reason behind the insignificant association of employment next to the study and mental health indicators. Further differentiation in the type of work of students employed next to their study (i.e., veterinary-related, non-veterinary-related) was not done and should be considered in future studies. This might help in the differentiation between students who work due to financial issues or those working in the veterinary field primarily to gain further experience and training in the profession.

This study has several shortcomings that need to be considered when interpreting the current findings.

As the study was conducted cross-sectionally, cause-and-effect relationships are not possible to examine. Further, longitudinal studies are required to elucidate the specific mental health trends and predictors during veterinary medical education and work in the veterinary profession. Although the study had a high response rate, there might be some bias toward the participation of more burdened students. No information on potential previous mental illnesses was collected. Also, the investigated mental health indicators rely solely on self-reports and have not been confirmed by clinical interviews. Additionally, health behaviors were not assessed objectively, but rather by self-reports. Further limitations are missing pieces of information on several study-related variables, such as academic performance, current stressors (i.e., exam phases), or potential financial concerns. The moderate internal consistency of the CAGE and SCOFF scales are additional limitations.

## Conclusion

Results on poor mental health in Austrian veterinary students are concerning since high levels of depression and anxiety can continue in practicing veterinarians which have been shown to have higher suicide rates than the general public. Overall, the results highlight the need to promote mental health in veterinary students. Especially female students and those of older age might benefit from specific support to deal with the academic workload and other stressors. Veterinary universities may wish to consider the merits of integrating personal life balance training in veterinary programs. The provision of training in dealing with study-related distress, anxiety, and depression might help not only improve the mental well-being of veterinary students but also their academic performance and reduce drop-out rates.

## Methods

### Design

Students of veterinary medicine at the University of Veterinary Medicine Vienna, the only veterinary educational institution in Austria, were surveyed online between November 28, 2022, and January 31, 2023, using LimeSurvey (LimeSurvey GmbH, Hamburg, Germany). The study was conducted in the third year of the COVID-19 pandemic. During the time of the survey, only minimal COVID-19 restrictions were in place, such as mask mandates in healthcare facilities and mobility restrictions of COVID-19 infected individuals^31^. The Union of Students of the University of Veterinary Medicine Vienna as well as the registrar’s office of the University of Veterinary Medicine Vienna invited all students enrolled in the diploma study of veterinary medicine to participate (1477 students, 1213 females (82.1%), 264 males). Students received no incentives and participation was voluntary and anonymous. They had to fill out the survey once in this cross-sectional study.

This survey was carried out according to the Declaration of Helsinki. The data protection officer as well as the Ethics Committee of the University for Continuing Education Krems, Austria, approved the study (Ethical number: EK GZ 25/2021-2024). To take part in the survey, all students had to provide electronic informed consent.

To compare the mental health indicators of veterinary students with the Austrian general population, data collected on a representative sample of the Austrian general population in April 2022 were used. The study was conducted in the third year of the COVID-19 pandemic. The spring of 2022 was characterized by the relaxation of COVID-19 measures. More specifically, containment efforts in April 2022 were mainly limited to the need to wear face masks in critical infrastructures, compulsory segregation of COVID-19-infected individuals, and the requirement of proof of low epidemic risk (i.e., proof to be vaccinated, recovered, or tested) upon entering Austria^30^. A few weeks before the survey of the general population several new crises and issues emerged (i.e., the Russian-Ukraine war and a gas/energy crisis)^31^.

Recruitment and study design have been reported in detail^26^. In brief, quota sampling was utilized to select a total of N = 1011 adult participants, based on predefined quotas for crucial demographic factors, such as age, sex, age × sex, region, and educational level. A comprehensive description of the quota sampling categories, intended quotas (derived from data provided by the Austrian Federal Statistical Office), and the corresponding final quotas achieved has been presented in detail in our companion paper^26^. All sociodemographic data, mental health indicators, and health behaviors reported in the following were assessed in the general population sample using the same questions.

### Measures

All measures are presented in detail in Suppl. Table S7.

#### Sociodemographic variables

Students were asked about their gender (female, male, diverse), age (in years), country of origin (Austria, foreign countries), and partnership status (single, partnership).

#### Health behaviors 

As health behaviors smartphone use (< 1 h/day, 1–2 h/day, 3–4 h/day, 5–6 h/day, 7–8 h/day, > 8 h/day) and physical activity (days of at least 60 min of physical activity per week) were assessed by self-report. Following recent recommendations to exercise at least 3 days per week^37^, physical activity was divided into two groups (≤ 2 days of physical activity per week compared to ≥ 3 days of physical activity per week). Similarly, smartphone usage was grouped into students using the smartphone for at least 3 h per day compared to those spending up to 2 h per day on their smartphone based on current data on the association of smartphone use and mental health^38^.

#### Study-related variables

Participants were further asked about the semester of study they were in. The study phase was assessed following the curricula of the diploma study of veterinary medicine as follows: 1st study phase (basic knowledge about the living organism, 1st–4th semester), 2nd study phase (deepened knowledge about physiological and pathological processes, 5th–9th semester), 3rd study phase (specification, 10th–12th semester). Moreover, the survey assessed whether they engage in a professional activity next to their study, and if yes for how many hours per week, their preferred specialization (small animal medicine; ruminant medicine; poultry and swine medicine; reproductive technology; equine medicine; conservation medicine (zoo and wildlife medicine); laboratory animal medicine; food science, public veterinary, and health care). The curriculum in the 3^rd^ study phase differs among specifications. Since the students in the first two study phases were not yet in a specialization, the question asked about the preferred specification, so that all students felt addressed. The specifications in poultry and swine medicine, reproductive technology, laboratory animal medicine as well as food science, public veterinary, and health care were summarized as “others” for statistical analyses due to the low number of students per single category.

#### Indicators of mental health

To screen for mental health disorders standardized screening tools validated in German have been applied. These scales have been originally developed to screen for mental disorders in clinical settings, mainly based on the diagnostic criteria described in the Diagnostic and Statistical Manual of Mental Disorders (DSM)^39,40^. These screening tools are used in clinical practice and research as they provide efficient diagnostic tools for common mental health disorders. Screening tools for mental health disorders provide valuable insights, but it is important to note that no diagnosis can be based on solely exceeding the respective cut-offs until confirmed by clinical interviews conducted by trained professionals (i.e., the structured clinical interviews for the DSM, the “SCID”). Due to the online nature of the study and the strictly anonymous data collection, no clinical interviews were possible. Therefore, we refer to “symptoms of mental health disorders” throughout the paper.

#### Perceived stress (PSS-4)

The subjective stress levels over the past two weeks were measured with the German version of the perceived Stress Scale^41^. The PSS-4 represents a self-report tool to assess perceived stress on a five-point Likert scale from 0 (never) to 4 (very often)^42^. Total scores are calculated by summing up the scores of all items after reversed coding of items 2 and 3, yielding scores from 0 to 16. Higher scores are indicative of a higher subjective stress level, with a cut-off point of at least 6 points being considered high stress levels^43^. Internal consistency (Cronbach's alpha) was α = 0.82 in the present students' sample.

#### Depressive symptoms (PHQ-9)

Symptoms of depression during the past two weeks were evaluated with the German version of the nine self-rating items of the depression module of the Patient Health Questionnaire^44^. The PHQ-9 assesses depressive symptoms on a four-point scale from 0 (not at all) to 3 (nearly every day). The total score ranges from 0 to 27, with higher scores indicating more severe depressive symptoms. Scores ≥ 10 indicate clinically relevant depressive symptoms^45^. Cronbach's alpha was α = 0.84.

#### Anxiety (GAD-7)

The seven items of the General Anxiety Disorder scale^46^ validated in German^47^ were applied to measure symptoms of anxiety over the last two weeks. The seven self-rating items of the GAD-7 scale assess symptoms of generalized anxiety on a four-point Likert scale from 0 (not at all) to 3 (nearly every day), yielding sum scores from 0 to 21. Total scores of at least 10 points are considered as indicating moderate (clinically relevant) anxiety symptoms^47^. Cronbach's alpha was α = 0.88 in the present sample.

#### Insomnia (ISI-7)

The Insomnia Severity Index (ISI) was applied to assess sleep quality^48^. The German version of the scale was used^49^. The seven self-rating items of the ISI-7 measure are rated on a five-point scale from 0 to 4. The total score ranges from 0 to 28, with a cut-off score of ≥ 15 indicating clinically relevant insomnia symptoms. Cronbach's alpha was α = 0.81.

#### Alcohol abuse (CAGE)

Symptoms of alcohol abuse were assessed with the German version of the CAGE questionnaire^50,51^. The CAGE comprises four yes/no questions asking about signs of alcoholism, such as questions about Cutting down, Annoyance with criticism, feelings of Guilt, and Eye-openers. The sum scores range from 0 to 4, with scores of at least 2 points being indicative of alcohol abuse^52^. Cronbach's alpha was α = 0.56 in the present students' sample.

#### Disordered eating (SCOFF)

Symptoms of disordered eating were measured with the German version of the SCOFF questionnaire^53,54^. The SCOFF comprises five yes/no questions asking about signs of anorexia nervosa and bulimia nervosa, such as questions about making oneself feel Sick because of feeling uncomfortably full, losing Control while eating, losing more than One stone in 3 months, concerns about being too Fat and dominance of Food in one’s life. The sum scores range from 0 to 5, with scores of at least 2 points being indicative of disordered eating^55^. Cronbach's alpha was α = 0.48 in the present students' sample.

### Sample size

Power analysis for univariate analyses was performed with G ∗ Power 3.1.9.4^56^(45). With an error type 1 of 0.05, and a power of 0.80 and an expected effect size of w = 0.2, a total sample size of 359 persons is required when the use of Chi-squared-tests. The degrees of freedom were set at 7 to calculate the sample size for the comparison with the most groups (i.e., the desired specification).

For the multivariable analyses the minimum sample size has been calculated based on recommendations to reduce the risk of overfitting (Type I error) or underfitting (Type II error) by including at least 20 cases per predictor^57^. As we investigated 9 predictors, we aimed to recruit at least 180 participants to reduce the risk of overfitting (Type I error) or underfitting (Type II error).

Assuming a response rate of 25%^58^, the total population of veterinary students (n = 1477) was invited to participate.

### Statistical analyses

Descriptive statistics were conducted to describe sociodemographic characteristics.

As the low number of students describing themselves as gender-diverse (n = 3) did not allow for statistical analyses, these participants were excluded from further statistical analyses.

To compare mental health indicators in veterinary students with the general population, Chi-squared tests were conducted. To account for differences in age and gender between both samples, multivariable binary logistic regressions were run with the mental health indicators as dependent variables and group (veterinary students compared to general population), gender, and age as predictors.

Chi-squared tests were conducted to analyze differences in the prevalence of symptoms of clinically relevant depression, anxiety, insomnia, stress, alcohol abuse, and eating disorders regarding sociodemographic characteristics (age, gender, country of origin, partnership status), health behaviors (physical activity, smartphone usage) and study-related variables (employment, study phase, intended specification). To explore the independent contribution of the investigated sociodemographic variables, health behaviors, and study-related factors associated with clinically relevant mental health symptoms in veterinary medicine students, multivariable binary logistic regression was used. The mental health variables served as dependent variables and the above-described sociodemographic variables, health behavior variables, and study-related variables as predictors.

For the binary logistic regression analyses, adjusted odds ratios (aOR) and their 95% confidence intervals (CIs) were estimated to assess the statistical uncertainty. Bonferroni correction was used for post-hoc tests following significant chi-squared tests. Statistical analyses were performed using SPSS version 26 (IBM Corp, Armonk, NY, USA). *P*-values of < 0.05 were considered statistically significant (2-sided tests).

### Supplementary Information

Supplementary Tables.

## Data Availability

The datasets used during the current study are available from the corresponding author on reasonable request.
