# Supplementary material for: Prevalence of mental health symptoms and potential risk factors among Austrian veterinary medicine students
Source: Sci Rep. 2023 Aug 23;13:13764. doi: 10.1038/s41598-023-40885-0 (PMC10447431; doi:10.1038/s41598-023-40885-0)
Supplement: Supplementary file 1 — Supplementary Tables. [file 41598_2023_40885_MOESM1_ESM.docx]

**Suppl. Table 1** Results of binary logistic regression analyses for depressive symptoms

| Variable | aOR | 95% CI | | *P*-Value |
| --- | --- | --- | --- | --- |
| Gender  (male vs female) | .835 | .446 | 1.564 | .574 |
| **Age** | **1.133** | **1.045** | **1.228** | **.002** |
| Partnership status (partnership vs single) | 1.219 | .795 | 1.870 | .364 |
| Country (Not Austria vs Austria) | 1.147 | .736 | 1.788 | .544 |
| Study phase |  |  |  | .804 |
| (second vs first) | 1.036 | .628 | 1.708 | .890 |
| (third vs first) | .846 | .431 | 1.661 | .626 |
| Job (no vs yes) | 1.123 | .720 | 1.753 | .609 |
| Intended specialization |  |  |  | .000 |
| **(ruminant vs small animal medicine)** | **.420** | **.233** | **.760** | **.004** |
| **(equine vs small animal medicine)** | **.427** | **.243** | **.752** | **.003** |
| (conservation vs small animal medicine) | 1.640 | .862 | 3.119 | .132 |
| (others vs small animal medicine) | .662 | .296 | 1.483 | .316 |
| Physical activity  (≥3d/wk vs ≤2d/wk) | .689 | .457 | 1.040 | .076 |
| Smartphone usage (≥3h/d vs ≤2h/d) | 1.428 | .918 | 2.221 | .114 |

**Suppl. Table 2** Results of binary logistic regression analyses for anxiety symptoms

| Variable | aOR | 95% CI | | *P*-Value |
| --- | --- | --- | --- | --- |
| **Gender  (male vs female)** | **.435** | **.227** | **.834** | **.012** |
| **Age** | **1.084** | **1.010** | **1.164** | **.026** |
| Partnership status (partnership vs single) | 1.475 | .962 | 2.263 | .075 |
| Country (Not Austria vs Austria) | 1.106 | .710 | 1.724 | .655 |
| Study phase |  |  |  | .963 |
| (second vs first) | 1.009 | .615 | 1.655 | .972 |
| (third vs first) | .929 | .481 | 1.794 | .826 |
| Job (no vs yes) | 1.531 | .979 | 2.394 | .062 |
| Intended specialization |  |  |  | .007 |
| **(ruminant vs small animal medicine)** | **.459** | **.252** | **.837** | **.011** |
| **(equine vs small animal medicine)** | **.515** | **.292** | **.906** | **.021** |
| (conservation vs small animal medicine) | 1.380 | .737 | 2.584 | .314 |
| (others vs small animal medicine) | .589 | .260 | 1.338 | .206 |
| **Physical activity  (≥3d/wk vs ≤2d/wk)** | **.559** | **.370** | **.844** | **.006** |
| Smartphone usage (≥3h/d vs ≤2h/d) | 1.324 | .850 | 2.061 | .215 |

**Suppl. Table 3** Results of binary logistic regression analyses for insomnia symptoms

| Variable | aOR | 95% CI | | *P*-Value |
| --- | --- | --- | --- | --- |
| Gender  (male vs female) | 1.064 | .506 | 2.235 | .871 |
| Age | 1.069 | .996 | 1.146 | .065 |
| Partnership status (partnership vs single) | 1.198 | .722 | 1.988 | .483 |
| Country (Not Austria vs Austria) | 1.202 | .721 | 2.004 | .481 |
| Study phase |  |  |  | .804 |
| (second vs first) | 1.075 | .601 | 1.924 | .807 |
| (third vs first) | .858 | .407 | 1.807 | .686 |
| Job (no vs yes) | .800 | .477 | 1.342 | .398 |
| Intended specialization |  |  |  | .281 |
| (ruminant vs small animal medicine) | .533 | .253 | 1.123 | .098 |
| (equine vs small animal medicine) | .513 | .249 | 1.055 | .069 |
| (conservation vs small animal medicine) | .754 | .367 | 1.550 | .442 |
| (others vs small animal medicine) | .755 | .291 | 1.956 | .562 |
| Physical activity  (≥3d/wk vs ≤2d/wk) | .965 | .593 | 1.569 | .885 |
| Smartphone usage (≥3h/d vs ≤2h/d) | 1.133 | .673 | 1.908 | .639 |

**Suppl. Table 4** Results of binary logistic regression analyses for high-stress symptoms

| Variable | aOR | 95% CI | | *P*-Value |
| --- | --- | --- | --- | --- |
| Gender  (male vs female) | .963 | .466 | 1.990 | .920 |
| Age | 1.038 | .954 | 1.130 | .386 |
| Partnership status (partnership vs single) | .877 | .528 | 1.456 | .612 |
| Country (Not Austria vs Austria) | .736 | .439 | 1.234 | .245 |
| Study phase |  |  |  | .333 |
| (second vs first) | 1.405 | .780 | 2.529 | .257 |
| (third vs first) | 1.766 | .788 | 3.956 | .167 |
| Job (no vs yes) | 1.307 | .770 | 2.220 | .322 |
| Intended specialization |  |  |  | .121 |
| (ruminant vs small animal medicine) | .670 | .340 | 1.319 | .247 |
| (equine vs small animal medicine) | .983 | .495 | 1.954 | .961 |
| (conservation vs small animal medicine) | 1.639 | .711 | 3.777 | .246 |
| **(others vs small animal medicine)** | **.421** | **.179** | **.991** | **.048** |
| Physical activity  (≥3d/wk vs ≤2d/wk) | .744 | .456 | 1.215 | .238 |
| Smartphone usage (≥3h/d vs ≤2h/d) | 1.301 | .775 | 2.183 | .320 |

**Suppl. Table 5** Results of binary logistic regression analyses for symptoms of disordered eating

| Variable | aOR | 95% CI | | *P*-Value |
| --- | --- | --- | --- | --- |
| **Gender  (male vs female)** | **.482** | **.240** | **.966** | **.040** |
| Age | .993 | .928 | 1.062 | .831 |
| Partnership status (partnership vs single) | .869 | .568 | 1.331 | .519 |
| Country (Not Austria vs Austria) | .972 | .628 | 1.504 | .897 |
| Study phase |  |  |  | .570 |
| (second vs first) | 1.185 | .722 | 1.945 | .502 |
| (third vs first) | 1.410 | .741 | 2.684 | .296 |
| Job (no vs yes) | .849 | .546 | 1.319 | .466 |
| Intended specialization |  |  |  | .310 |
| (ruminant vs small animal medicine) | .618 | .330 | 1.157 | .133 |
| (equine vs small animal medicine) | 1.091 | .628 | 1.896 | .758 |
| (conservation vs small animal medicine) | 1.209 | .662 | 2.208 | .536 |
| (others vs small animal medicine) | 1.501 | .670 | 3.361 | .324 |
| Physical activity  (≥3d/wk vs ≤2d/wk) | 1.059 | .703 | 1.596 | .782 |
| **Smartphone usage (≥3h/d vs ≤2h/d)** | **1.854** | **1.180** | **2.912** | **.007** |

**Suppl. Table 6** Results of binary logistic regression analyses for symptoms of alcohol abuse

| Variable | aOR | 95% CI | | *P* |
| --- | --- | --- | --- | --- |
| **Gender  (male vs female)** | **3.075** | **1.593** | **5.935** | **.001** |
| Age | 1.018 | .943 | 1.098 | .652 |
| Partnership status (partnership vs single) | 1.428 | .858 | 2.378 | .170 |
| Country (Not Austria vs Austria) | .968 | .575 | 1.630 | .903 |
| Study phase |  |  |  | .196 |
| (second vs first) | 1.143 | .625 | 2.089 | .664 |
| (third vs first) | 1.882 | .915 | 3.870 | .086 |
| Job (no vs yes) | .978 | .580 | 1.647 | .932 |
| Intended specialization |  |  |  | .819 |
| (ruminant vs small animal medicine) | 1.429 | .725 | 2.815 | .302 |
| (equine vs small animal medicine) | 1.145 | .571 | 2.298 | .703 |
| (conservation vs small animal medicine) | 1.407 | .689 | 2.872 | .349 |
| (others vs small animal medicine) | 1.252 | .498 | 3.147 | .633 |
| **Physical activity  (≥3d/wk vs ≤2d/wk)** | **.417** | **.254** | **.687** | **.001** |
| Smartphone usage (≥3h/d vs ≤2h/d) | 1.016 | .604 | 1.709 | .951 |

**Suppl. Table 7** Measures included in the survey on mental health in veterinary students

| The questions in this scale ask you about your feelings and thoughts during the last 2 weeks. | Never | Almost never | Sometimes | Fairly often | Very often |
| --- | --- | --- | --- | --- | --- |
| In the last 2 weeks, how often have you felt that you were unable to control the important things in your life? | 🞏 | 🞏 | 🞏 | 🞏 | 🞏 |
| In the last 2 weeks, how often have you felt confident about your ability to handle your personal problems? | 🞏 | 🞏 | 🞏 | 🞏 | 🞏 |
| In the last 2 weeks, how often have you felt that things were going your way? | 🞏 | 🞏 | 🞏 | 🞏 | 🞏 |
| In the last 2 weeks, how often have you felt difficulties were piling up so high that you could not overcome them? | 🞏 | 🞏 | 🞏 | 🞏 | 🞏 |

| Please rate the current (i.e., last 2 weeks) severity of your insomnia problem(s). | None | Mild | Moderate | Severe | Very severe |
| --- | --- | --- | --- | --- | --- |
| Difficulty falling asleep | 🞏 | 🞏 | 🞏 | 🞏 | 🞏 |
| Difficulty staying asleep | 🞏 | 🞏 | 🞏 | 🞏 | 🞏 |
| Problems waking up too early | 🞏 | 🞏 | 🞏 | 🞏 | 🞏 |

| How satisfied/dissatisfied are you with your current sleep pattern? | Very satisfied | Satisfied | Moderately satisfied | Dissatisfied | Very dissatisfied |
| --- | --- | --- | --- | --- | --- |
|  | 🞏 | 🞏 | 🞏 | 🞏 | 🞏 |

| How noticeable to others do you think your sleep problem is in terms of impairing the quality of your life? | | Not at all noticeable | A little | | Somewhat | | Much | | Very much noticeable | |
| --- | --- | --- | --- | --- | --- | --- | --- | --- | --- | --- |
|  | | 🞏 | 🞏 | | 🞏 | | 🞏 | | 🞏 | |
| How worried/distressed are you about your current sleep problem? | Not at all | | | A little | | Somewhat | | Much | | Very much |
|  | 🞏 | | | 🞏 | | 🞏 | | 🞏 | | 🞏 |

| To what extent do you consider your sleep problem to interfere with your daily functioning (e.g., daytime fatigue, mood, ability to function at work/daily chores, concentration, memory, mood, etc.)? | Not at all interfering | A little | Somewhat | Much | Very much interfering |
| --- | --- | --- | --- | --- | --- |
|  | 🞏 | 🞏 | 🞏 | 🞏 | 🞏 |

| On how many of the last 7 days were you physically active for at least 60 minutes?? | On 0 days | On 1 day | On 2 days | On 3 days | On 4 days | On 5 days | On 6 days | On 7 days |
| --- | --- | --- | --- | --- | --- | --- | --- | --- |
|  | 🞏 | 🞏 | 🞏 | 🞏 | 🞏 | 🞏 | 🞏 | 🞏 |

| In a typical day, how much time do you spend - sitting or lying down - on your cell phone? | Less than 1 hr/day | 1 to 2 h / d | 3 to 4 h / d | 5 to 6 h / d | 7 to 8 h / d | more than 8 hrs/day |
| --- | --- | --- | --- | --- | --- | --- |
|  | 🞏 | 🞏 | 🞏 | 🞏 | 🞏 | 🞏 |

| Have you ever... | Yes | No |
| --- | --- | --- |
| felt the need to cut down your drinking | 🞏 | 🞏 |
| felt annoyed by criticism of your drinking | 🞏 | 🞏 |
| had guilty feelings about drinking | 🞏 | 🞏 |
| taken a morning eye opener? | 🞏 | 🞏 |

|  | Yes | No |
| --- | --- | --- |
| Do you make yourself sick because you feel uncomfortably full? | 🞏 | 🞏 |
| Do you worry that you have lost control over how much you eat? | 🞏 | 🞏 |
| Have you recently lost more than one stone (14 lb) in a 3-month period? | 🞏 | 🞏 |
| Do you believe yourself to be fat when others say you are too thin? | 🞏 | 🞏 |
| Would you say that food dominates your life? | 🞏 | 🞏 |

| Over the last 2 weeks, how often have you been bothered by the following problems? | Not at all | Several days | More than half the days | Nearly every day |
| --- | --- | --- | --- | --- |
| Feeling nervous, anxious, or on edge | 🞏 | 🞏 | 🞏 | 🞏 |
| Not being able to stop or control worrying | 🞏 | 🞏 | 🞏 | 🞏 |
| Worrying too much about different things | 🞏 | 🞏 | 🞏 | 🞏 |
| Trouble relaxing | 🞏 | 🞏 | 🞏 | 🞏 |
| Being so restless that it's hard to sit still | 🞏 | 🞏 | 🞏 | 🞏 |
| Becoming easily annoyed or irritable | 🞏 | 🞏 | 🞏 | 🞏 |
| Feeling afraid as if something awful might happen | 🞏 | 🞏 | 🞏 | 🞏 |

| Over the last 2 weeks, how often have you been bothered by the following problems? | Not at all | Several days | More than half the days | Nearly every day |
| --- | --- | --- | --- | --- |
| Little interest or pleasure in doing things | 🞏 | 🞏 | 🞏 | 🞏 |
| Feeling down, depressed or hopeless | 🞏 | 🞏 | 🞏 | 🞏 |
| Trouble falling asleep, staying asleep, or sleeping too much | 🞏 | 🞏 | 🞏 | 🞏 |
| Feeling tired or having little energy | 🞏 | 🞏 | 🞏 | 🞏 |
| Poor appetite or overeating | 🞏 | 🞏 | 🞏 | 🞏 |
| Feeling bad about yourself - or that you’re a failure or have let yourself or your family down | 🞏 | 🞏 | 🞏 | 🞏 |
| Trouble concentrating on things, such as reading the newspaper or watching television | 🞏 | 🞏 | 🞏 | 🞏 |
| Moving or speaking so slowly that other people could have noticed. Or, the opposite - being so fidgety or restless that you have been moving around a lot more than usual | 🞏 | 🞏 | 🞏 | 🞏 |

Finally, please answer a few general questions about yourself and your professional activities for statistical purposes

| Please specify your gender | Female | Male | Diverse |
| --- | --- | --- | --- |
|  | 🞏 | 🞏 | 🞏 |

| Please enter your age in years (e.g. 24) |
| --- |
|  |

| What is your marital status? |  |
| --- | --- |
| Single | 🞏 |
| In relationship/partnership | 🞏 |

| Where were you born? |  |
| --- | --- |
| Upper Austria | 🞏 |
| Lower Austria | 🞏 |
| Vienna | 🞏 |
| Styria | 🞏 |
| Burgenland | 🞏 |
| Carinthia | 🞏 |
| Salzburg | 🞏 |
| Tyrol | 🞏 |
| Vorarlberg | 🞏 |
| I was not born in Austria, but in: | 🞏 |
|  | |

| In which semester are you studying veterinary medicine? |
| --- |
|  |

| Are you working alongside your studies? | Yes | No |
| --- | --- | --- |
|  | 🞏 | 🞏 |

| On average, how many hours per week do you spend in a professional activity? |
| --- |
|  |

| Which field of veterinary medicine interests you the most? |  |
| --- | --- |
| Small animal medicine | 🞏 |
| Ruminant medicine | 🞏 |
| Poultry and swine medicine | 🞏 |
| Reproductive technology | 🞏 |
| Equine Medicine | 🞏 |
| Conservation medicine (zoo and wildlife medicine) | 🞏 |
| Laboratory animal medicine | 🞏 |
| Food science, public veterinary, and health care | 🞏 |
